# Peer Support in the Treatment of Chronic Pain in Adolescents: A Review of the Literature and Available Resources

**DOI:** 10.3390/children7090129

**Published:** 2020-09-07

**Authors:** James A. Tolley, Marti A. Michel, Amy E. Williams, Janelle S. Renschler

**Affiliations:** 1 Department of Anesthesia, 1130 West Michigan St., Fesler Hall 204, Indianapolis, IN 46202, USA; mmichel@iuhealth.org (M.A.M.); jsrensch@iu.edu (J.S.R.); 2 Department of Psychology, Indiana University School of Medicine, Riley Hospital for Children, 705 Riley Hospital Drive, Indianapolis, IN 46202, USA; amyewill@iupui.edu

**Keywords:** chronic pain, peers, support, social network, virtual, internet, fibromyalgia, complex regional pain syndrome

## Abstract

Peer support has found applications beyond the mental health field and is useful for managing several chronic disorders and supporting healthy lifestyle choices. Communication through telephone and the Internet allows for greater access to those who cannot meet in person. Adolescent chronic pain would seem ideally suited to benefit from online peer support groups. Research is lacking, however, to characterize benefit in terms of pain and function, despite a clear desire among adolescents for access to such programs. More rapid development of online applications is needed for peer support, and research into the associated outcomes will be necessary to optimally design such programs.

## 1. Introduction

The concept of using peer support as a modality for the treatment of disease began in France in the late 1700s. Jean Baptiste Pussin, a hospital superintendent in Paris, described his rationale for hiring staff from among those who had recovered from mental illness. He stated that he found these individuals to be less cruel and kinder, exhibiting gentleness and honesty such that the hospital was able to eliminate the need for physical restraints and abuse of those suffering from severe mental illness [1].

The value of peer support was recognized and introduced into the United States by Harry Stack Sullivan in 1920s Baltimore, where he recruited recovering young men to work as aides in his inpatient mental facility [2]. The idea was further expanded when Alcoholics Anonymous, founded in 1935, demonstrated the effectiveness of self-help compared with the treatments utilized by the medical community experts at the time [2].

Peer support remained in the purview of mental health and addiction treatment until the latter half of the twentieth century, when groups for cancer, HIV/AIDS, diabetes, and other chronic disorders began to organize [3]. A 1999 report by the United States Surgeon General on mental health confirmed the efficacy of peer groups in the treatment of those with mental illness [2]. Today, peer support groups have expanded to include the promotion of healthy behaviors such as smoking cessation [4], weight loss [5], and breastfeeding [6].

Until relatively recently, one of the limitations of peer support groups was the necessity to attend meetings in person. The advent of the personal computer and the Internet paved the way for online bulletin boards, blogging, and social networking sites for health information and peer support. The rapid expansion of internet access from 413 million users in the year 2000 to an estimated 4.8 billion users in the second quarter of 2020 [7,8] has led to online peer support groups for many of the same conditions with in-person support groups.

When analyzing the literature on peer support for mental illness treatment, Davidson suggested a continuum of three stages [1]. The first stage—feasibility—is designed to determine if it is possible to train and hire prior patients to serve as staff members. The second stage is to assess the ability of these trained peers to deliver services compared with non-peer staff. Finally, the third stage is to determine if there is any additional benefit in treatment when peers are involved versus conventional therapy with non-peer providers.

Chronic pain—defined as pain lasting longer than 3 months—is common in adolescents. Epidemiological studies show a chronic pain prevalence of 15–30% in young people, with the head, abdomen, and limbs as the most common sites [9,10,11,12]. This pain has been described as distressing and interfering, sometimes severely debilitating, and affecting all aspects of the young person’s life (including parents and family members) [13,14]. The effects of adolescent pain may also extend into adulthood [15,16].

Adolescents with chronic pain are more isolated than healthy peers, are felt to be less likable, and, as a result, are more apt to be victims of bullying and have fewer friends [17,18]. Children and adolescents with chronic pain may have fewer opportunities to develop strong peer relationships due to school absence or avoidance of normative activities. In a study of children and adolescents with chronic pain and restrictions in daily living, 48.8% of participants reported missing school (35.8% “sometimes” and 13% “often or always”), and 46.7% cancelled plans with friends (32.9% “sometimes” and 13.8% “often or always”) [19]. Furthermore, healthy peers are less likely to be empathetic toward an individual in pain when the cause is not clearly organic, which is often the case in chronic pain disorders [20]. The net effect is that more than half of adolescents experiencing chronic pain judge themselves to be less developed than their healthy peers in terms of “independence, emotional adjustment, and identity formation” [21]. On the other hand, strong peer support had a positive impact on these three areas of adolescent development. In the presence of adaptive parenting, high-quality relationships with peers can best support the resiliency of adolescents with chronic pain, potentially leading to better functional and pain outcomes [22]. 

Chronic pain providers (physicians, psychologists, social workers, and a nurse) reported in structured interviews that adolescents would benefit from peer support if they had good peer relationships prior to the onset of disease and they were comfortable in sharing about their pain [23]. Such benefits might include a decreased sense of isolation, interactive empathy and feedback, treatment techniques, and an improved sense of community. Adolescents with chronic disease reported interest in developing self-management skills through mentor programs. They felt that flexibility in the delivery (e.g., internet, in-person, Skype calls, or text messaging) was important as were the flexibility of the content, frequency of interactions, and length of the program [24]. Potential concerns raised were the incompatibility of mentors and mentees as well as scheduling conflicts.

In considering online peer support groups for adolescent chronic pain, we would modify Davidson’s stages. The existence of these groups should first be considered along with any formal research into the feasibility of training adolescents or young adults to serve as mentors or facilitators of these groups. Next, we would examine any research on the ability of these groups to deliver peer support without causing harm compared with conventional therapies. Finally, we would seek to determine if there is any research that would suggest improved outcomes over conventional treatments as provided by professionals.

## 2. Feasibility

Many different platforms are now available for peer support for adolescent chronic pain (Table 1). These include social media groups (e.g., Facebook), video platforms (e.g., YouTube), smartphone apps, and other unique programs. Organizations such as The Coalition Against Pediatric Pain and the U.S. Pain Foundation also offer some support programs such as a pediatric pain camp [25] and an ambassador network [26].

Facebook is the most widely used social networking site, with a reported 2.5 billion users [36]. A 2018 study found that 11 of 11 hospitalized adolescents used Facebook for entertainment and maintaining peer relationships while admitted for various reasons such as asthmatic attacks, dengue fever, and surgery [37]. When searching for potential adolescent peer support groups on Facebook, groups can be identified for teens with fibromyalgia [38,39], complex regional pain syndrome (CRPS) [40,41], Ehler–Danlos syndrome (EDS) [42,43,44], and sickle cell anemia [45], to name a few examples. Instagram (with over 1 billion users [46]) and Twitter (with over 330 million users [47]) are also social media platforms that would enable adolescents with chronic pain to interact and obtain health-related information.

A scoping review of YouTube video searching for the terms “youth with chronic pain” and “teens with chronic pain” yielded 18 videos and 936 viewer comments [48]. Viewers can connect with each other through the viewer comment section. Qualitative analysis of the video content was performed and covered multidisciplinary treatment options for various chronic pain conditions, alternative treatments, and the impact of pain on daily functioning. Viewer comments were focused on providing and receiving support, opportunities for sharing suffering, and discussion of the impact on daily life and relationships. One drawback to YouTube, Facebook, and other social media groups is a lack of supervision and content monitoring.

Kohut et al. looked at the feasibility of creating a peer mentorship program for young adults aged 18–25 who had successfully learned to manage chronic pain [27]. The mentors in this iPeer2Peer program were previous patients known to the health care team who were mature and emotionally stable, successfully self-managing their chronic pain, and had transitioned to an adult pain care model. Following 20 h of in-person training and self-directed training at home, these mentors interacted with younger adolescents via 10 Skype video calls over 8 weeks. The patients suffered from various causes of chronic pain including CRPS, chronic widespread pain, abdominal pain, and pain associated with chronic disease. This same group also examined the feasibility and efficacy of the iPeer2Peer intervention in adolescents with juvenile idiopathic arthritis (JIA) [28].

Allen et al. studied the feasibility and outcomes of matching trained adolescent mentors (aged 14–18 who had learned to successfully manage chronic pain) with similarly aged mentees to participate in a 2-month telephone-based peer-mentorship program [29]. Mentors would receive 6 h of training by a post-doctoral psychologist and then participate in ten 30–60 min phone calls during which information from a specifically designed slide presentation was to be discussed. Unfortunately, only 27 participants were enrolled with only 17 completing the study, and no updates have appeared in almost 4 years [29,30].

Smartphone apps can also be helpful to provide social support for adolescents suffering from chronic diseases that are associated with pain. The iManage mobile app was designed and tested in a collaborative effort with 70 adolescents and young adults (AYAs) with sickle cell anemia (SCA) [31]. These AYAs provided developers with information on internet access and mobile technology usage and helped to identify barriers to self-management, design an app to minimize those barriers, and test the feasibility and usability of a prototype. All of the AYAs felt that peer support would increase motivation in managing their disease.

Another app, iCanCope with Pain^TM^*,* was developed in conjunction with 23 adolescents recruited from chronic pain clinics in Canada [32]. In focus groups and individual interviews, the adolescents with pain reported that the peer support component was an important modality in the management of their pain. Health care providers also acknowledged the value that friendship with others experiencing chronic pain could provide in terms of encouragement. This application was also translated into the Norwegian language and culture, and its usability was tested [33] in a phased approach. End-user testing revealed only minor errors and that no major changes to the app were needed, except to facilitate user interaction in the social support component.

A Dutch group assessed the feasibility of developing both a face-to-face program and a web-based program led by peer leaders [34]. “Challenge your arthritis” was designed for adolescents and young adults aged 16–25 years of age with juvenile idiopathic arthritis (JIA). Trained peer leaders aged 20–30, who themselves had JIA, led participants through a weekly web-based chat lasting 90 min over a 6 week period. Each week covered a different theme such as self-efficacy, mood, activity, relationships, etc. The face-to-face program covered the same themes in a total of 12 h over 3 days at a resort. All participants in the online program found it “easy to use” and were pleased with the design of the site, and all 10 of them would recommend the program to others and would participate again. The plan upon completion of the feasibility study was to conduct a randomized controlled trial to assess patient outcomes.

## 3. Outcomes

There is a paucity of research into outcome measures for peer support in pediatric and adolescent chronic pain; therefore, drawing overall conclusions is difficult regarding lack of harm or increased efficacy when compared with conventional treatments. Anecdotal evidence has been reported, such as a case study for a telephone-based peer mentorship program in which a 19-year-old trained female mentor interacted with a 17-year-old male mentee [49]. After 10 weekly sessions, the mentee described improved sleep, self-esteem, and health perceptions as well as lower bodily pain.

This section will review the available outcomes research (Table 2) related to peer support and chronic pain in the child and teenager. Several studies have supported the benefits of integrating peers into in-person interventions for pain or chronic illness. Maslow and colleagues have evaluated the effectiveness of a peer mentoring group, The Adolescent Leadership Council, for adolescents with chronic illnesses including chronic pain [50]. This group includes college-aged peer mentors and utilizes a positive youth development approach to foster life skills and transition to adulthood. Twenty adolescents participating in this program completed pre- and post-assessments, which found decreased loneliness and increased health care self-advocacy after participation in the program.

Coakley et al. evaluated the effectiveness of a multi-family group-based intervention for children and adolescents with chronic pain [51]. This consisted of a single-day 6-hour group intervention with parents and children meeting in separate groups simultaneously. A peer mentor participated in a portion of the group as well. The results indicated that families liked the program, and preliminary evidence showed improvements in functioning, depression, and pain catastrophizing within 1–3 months after the intervention. The program also offers a virtual peer group “chat” for adolescents with chronic pain [55]. The individual components of this program (such as peer mentoring and virtual peer chat) could not be evaluated separately; therefore, it was not feasible to determine the contribution of peer support to overall improvements in the participants.

Arnstein and colleagues evaluated the transition from “chronic pain patient” to “peer volunteer” in the setting of a cognitive behavioral chronic pain management program to determine benefits and harms [52]. The study used a longitudinal, repeated-measures design using adult patients who had completed the pain-focused cognitive behavioral therapy program looking at quantitative and qualitative outcome measures. The patients served as their own controls as they transitioned from the patient phase to a training phase and then a volunteering phase. There were significant improvements in pain (Visual Analog Scale), disability (Pain Disability Index), and depression (Center for Epidemiological Study-Depression scale) during the transition from patient to peer. The Chronic Pain Self-Efficacy Scale was used to measure changes in self-efficacy related to pain management, coping, and physical function. There was a trend upward in self-efficacy scores during this same period, although these changes did not reach statistical significance. Peer volunteer interviews identified two themes—“making a connection” and “a sense of purpose”. There were no harms identified for the peer coaches. This study was limited by the small sample size. Subsequently, a larger, randomized exploratory study of peer-coaching outcomes in chronic pain failed to demonstrate benefit [53].

The outcome measures in the feasibility study of the iPeer2Peer program for individuals with JIA demonstrated that all the participants were satisfied with the program and would recommend it to other youths with JIA [28]. They enjoyed having the ability to communicate with a peer that had experienced issues that were both related and unrelated to JIA. The study also found that the participants scored higher on self-management scores compared with patients on a waitlist, but there was no significant difference with regard to pain scores or self-efficacy. The initial pain scores averaged less than 3 out of 10, and the sample size was small, which was a limitation that was recognized by the authors.

The other feasibility study of the iPeer2Peer program using a chronic pain population also looked at outcome measures [27]. Only 40% of the participants completed the program within the allotted time frame of 2 months, but all of them completed it once connected with a peer mentor. Again, all the participants would recommend the program to a friend with chronic pain. As in the previous study, self-management scores were significantly improved following the intervention. In addition, the ability to cope was judged to have improved by the participants. There was no difference in overall pain scores, even though initial pain scores were high (greater than 6 out of 10 on average).

No outcomes have yet been reported for the iCanCope with Pain app. As of 27 June 2020, the associated website [56] indicates that it is still a research study. Upon downloading the app on the Google Play Store, an access code or quick response code is needed to access the program, thus preventing the general public from using its features. It is unclear when or if the app would be available to the general public.

The Dutch developers of “Challenge your arthritis” completed the planned randomized controlled trial and published the results in 2017 [54]. A total of 72 patients with JIA were randomized into two equally sized groups. The study group participated in the online program in addition to usual care, consisting of routine follow-up appointments every 3 months in the transition clinic where treatment plans were discussed. The control group received usual care but were denied access to the online program until after the 6 month study follow-up period. No differences were found between the groups in self-efficacy as the primary outcome measure. Secondary outcome measures such as pain, quality of life, or absenteeism from work or school were also similar. Because study participants were recruited from a multi-disciplinary, tertiary care transition clinic with an emphasis on self-management, the authors suggested that this might explain the lack of benefit.

## 4. Discussion

Adolescence is a time during which teenagers increase their autonomy and develop a sense of personal and sexual identity, spending less time with parents and developing peer relationships [57]. However, the prevalence of chronic weekly pain over a 6 month period in adolescents can be as high as 44.2% (with an increasing incidence in females and older teenagers) [58], which can hamper the typical development processes that occur during this time. Unfortunately, this brief review of the literature did not find any randomized, controlled studies that actually demonstrated any objective improvements in either pain or function in adolescents as a result of online peer group support when compared with usual care. Participants in adolescent online support groups did receive some subjective benefits in terms of self-management and the feeling of improved coping with chronic pain. Moreover, all of them would have recommended such programs to friends with chronic pain. More research is needed to define the benefits of peer support in these patients and to determine the optimum platforms and programs.

In our chronic pain clinic, we often hear teenagers relate feelings of isolation, a lack of understanding by parents and friends as to the extent of their pain, and frustration with their bodies failing them. Even without the objective evidence showing benefit in terms of pain, perceived social support can have a positive impact on physical functioning and depression in adolescents and young adults with fibromyalgia [35]. Facebook groups might be useful in this situation. However, one of the problems with a group managed by an individual is waning interest as the adolescent ages and moves on to college, a career, and a family. This problem is apparent as some of the Facebook groups have had no posts in several years [38,40]. An alternative approach would be an online application or internet-based program that is sponsored by an institution such that mentors could be trained and replaced as they age or lose interest. Mentees could serve as a pool of potential future mentors as they learn and successfully implement disease self-management skills. The iCanCope with Pain app is a promising candidate but is still being studied.

Peer support programs for chronic pain might also be associated with certain limitations and/or weaknesses. These include a lack of content monitoring for some online groups, the potential for bullying, the normalization of maladaptive behaviors, confidentiality concerns, and inappropriate online medical advice. Online support groups should not be used as a replacement for in-person treatment programs; therefore, healthcare providers and parents should ensure that the adolescent also receives appropriate in-person therapy.

## 5. Conclusions

Peer support has been available to those with mental health disorders for over 200 years and has been an invaluable part of treatment for other chronic disorders. Until recently, peer support required meeting in person and was constrained by geography and time. With the invention of the Internet and smartphones, those barriers have been removed—allowing for billions to have access to health information, disease self-management skills, and support from peers. The COVID-19 pandemic and subsequent “stay at home” orders and quarantines have led to adolescents experiencing even more isolation and loneliness. Adolescents in chronic pain represent a unique patient population that is internet-savvy and who would appear to benefit from the ongoing development of social networks and applications in the treatment of their pain. More research is needed to provide objective evidence for the benefits of online peer support programs for these young people in chronic pain.

## Figures and Tables

**Table 1:** Available resources and feasibility studies of peer support for adolescents with chronic pain.

Type of Resource	Description	Reference
Social media		
Facebook	Interactive online groups available for various diseases, video posting with interaction in comments, etc.	Facebook.com
Instagram		Instagram.com
Twitter		Twitter.com
YouTube		YouTube.com
iPeer2Peer program	Peer mentorship for adolescents with chronic pain using Skype video chat	[27,28]
Peer mentorship program (Allen et al. [29])	Telephone-based peer support for teenagers with chronic pain	[29,30]
Smartphone apps		
iManage mobile app	App developed for supporting adolescents and young adults with sickle cell anemia	[31]
ICanCopeWithPain^TM^	App developed for adolescents with chronic pain in Canada and Norway	[32,33]
Challenge Your Arthritis program	Dutch program for young people with juvenile idiopathic arthritis, assessing both face-to-face and web-based support	[34]
The Coalition Against Pediatric Pain (TCAPP)	Pediatric summer camp and other support programs	[35]
The U.S. Pain Foundation	Ambassador Network and other programs (all ages)	[26]

**Table 2:** Outcomes research for peer support programs for adolescents with chronic pain.

Program or Study	Outcomes	Reference
The Adolescent Leadership Council (peer mentoring group for adolescents with chronic illness)	Decreased loneliness, increased health care self-advocacy.	[50]
The Comfort Ability multi-family group-based intervention for children and adolescents with chronic pain	Family approval for the program. Increased function and less depression and pain catastrophizing within 1–3 months.	[51]
Evaluation of transition from chronic pain patient to peer volunteer (risks and benefits)	Improved pain scores, depression, and disability. Peer volunteers reported “making a connection” and a “sense of purpose.” No harms were identified.	[52]
Randomized exploratory study of peer-coaching outcomes in chronic pain	No significant benefits for peer coaches compared with controls.	[53]
iPeer2Peer program evaluated for JIA	High satisfaction, increased self-management scores. No changes in pain scores or self-efficacy compared with JIA patients on a waitlist.	[28]
iPeer2Peer program evaluated in a chronic pain population	High satisfaction; improved self-management. Similar pain scores compared with pre-intervention scores.	[27]
Challenge Your Arthritis randomized controlled trial of 72 patients with JIA	Similar self-efficacy, pain scores, quality of life, and absenteeism from work/school between intervention and control groups.	[54]

JIA = juvenile idiopathic arthritis.
